# P34 - Food-induced anaphylaxis in children: most common triggers in the Czech Republic

**DOI:** 10.1186/2045-7022-4-S1-P89

**Published:** 2014-02-28

**Authors:** Simona Belohlavkova, Radka Kabrnova, Tereza Pospisilova, Martin Fuchs

**Affiliations:** 1 Prague General University Hospital, Prague, Czech Republic

## Introduction

Food is one of the most common causes of anaphylaxis responsible for more than 50-75% of anaphylactic cases in children. Most frequently implicated foods in childhood are milk, egg, nuts, sesame, fish and shellfish worldwide. There are some differences in incidence of types of food allergy in different areas. We tried to describe most common food anaphylactic triggers in patients of regional outpatient allergic clinic.

## Methods

139 patients with history of allergic reaction to food fulfilling diagnostic criteria for anaphylaxis were prospectively included. 83 of them were children. We recorded the type of food responsible for reaction, threshold doses eliciting the reaction, levels of specific IgE against food. We performed skin prick tests with native foods and in some patients also levels of specific IgE against components.

## Results

Not surprisingly, the most common triggers of food induced anaphylaxis in our children group were milk, peanut, tree nuts and egg (32%, 29%, 26% and 18%, resp.). Poppy seed, very uncommon cause of food allergy across the Europe, elicited food anaphylaxis in 14% of our children patients, usually with severe symptoms and low thresholds. In our group of adult patients, 25% patients with food anaphylaxis reacted to poppy seed.

## Discussion

Although poppy seed allergy is very rare around the world, it represents a common cause of food allergy and anaphylaxis in Czech Republic. Poppy seed allergens, described and included into allergen databases, are Pap s, Pap s 1 (Bet v 1 like), Pap s 2 (profilin) and Pap s 34. Allegic reaction to poppy seed are usually severe and patients react both to fresh and heated food. Therefore, most likely, poppy seed reactions are caused by some different, termostabile, proanaphylactic allergens (likely belonging to seed storage protein families), than Bet v 1 or profilin homologous allergens.

